# The effect of early warning scoring systems on adverse outcome in surgical patients: A systematic review

**DOI:** 10.1016/j.ijnsa.2024.100256

**Published:** 2024-10-28

**Authors:** Annick Stolze, Tara N.M. Woolley-Hendriks, Yara Bassa, Ralph de Vries, Christa Boer, Peter G. Noordzij

**Affiliations:** a Department of Anesthesiology, Amsterdam University Medical Centre, VU University, Amsterdam, The Netherlands; b Department of Anesthesiology, Intensive Care and Pain management, St. Antonius Hospital Nieuwegein, Nieuwegein, The Netherlands; c Medical Library, VU University, Amsterdam, The Netherlands

**Keywords:** Cardiopulmonary arrest, Clinical deterioration, Early Warning Score, Failure to rescue, Mortality, Outcome assessment, Patient acuity, Surgical patients, Systematic review

## Abstract

**Background:**

An early warning scoring system aims to detect clinical deterioration at an early stage and prevent failure-to-rescue in hospitalized patients. In this systematic review we studied the effect of an early warning scoring system on adverse outcome in surgical patients.

**Methods:**

This review was conducted and reported according to PRISMA and the protocol of this review is registered at PROSPERO, under the registration number CRD42018107799. PubMed, Embase.com, CINAHL (Ebsco) and Wiley/Cochrane Library were searched from inception up to 20-06-2023 for randomized controlled trials and non-randomized studies of interventions. Studies were eligible for inclusion if the effect of an early warning scoring system using spot check monitoring was studied.

**Results:**

Eight articles were included, of which two were randomised controlled trials. The overall risk of bias was high. A statistically significant decrease in mortality was seen in three studies. Two studies reported a decrease in cardiopulmonary arrests, and three studies found a decrease in ICU-admissions. There was heterogeneity among studies regarding the types of complications that were reported.

**Conclusions:**

The evidence in favor of an early warning scoring system to reduce complications and mortality in surgical patients is low, mainly due to a limited number of studies and poor study design. Well-designed trials are needed to investigate whether an early warning scoring system improves outcome in surgical patients.

## Contribution of the paper

**What is already known**

• early warning scoring systems aim to detect clinical deterioration at an early stage and prevent failure-to-rescue in hospitalized patients
• Early warning scoring systems have been implemented on patient wards/units over the last decades

Alt-text: Unlabelled box

**What this paper adds**

• The evidence in favor of early warning scoring systems to reduce complications and mortality in surgical patients is low; mainly due to a limited number of studies and poor study design
• Well-designed trials are needed to investigate whether an early warning scoring system improves outcome in surgical patients

Alt-text: Unlabelled box

## 1 Introduction

Postoperative complications are common in patients undergoing major surgery. In 2016, the International Surgical Outcomes Study reported an incidence of postoperative complications of >16 % (International Surgical Outcomes Study Group, 2016). The development of a postoperative complication has negative health consequences, such as a decrease in quality of life and a higher risk of mortality following a complication. The latter is also referred to as failure to rescue (Lafonte et al., 2019). In addition, postoperative complications increase health care costs substantially worldwide.

One way to improve perioperative care is to timely identify a patient that suffers a complication following surgery. A postoperative complication is often preceded by vital sign deterioration during hours before the event. As a result, early warning scoring systems have been developed and implemented on patient wards over the last decades. Early warning scoring systems are simple scoring systems where vital sign measurements are performed primarily every 6–12 h, also called spot check monitoring. An early warning scoring system aims to detect clinical deterioration and diagnose complications in a timely manner to prevent treatment delay in hospitalized patients (García-Del-Valle et al., 2021).

The prognostic value of early warning scoring systems has been studied extensively, but most studies have substantial limitations (Alhmoud et al., 2021). Besides, there is limited evidence that an early warning scoring system improves the outcome in surgical patients (García-Del-Valle et al., 2021). In this review we studied the effect of an early warning scoring system on adverse outcomes in surgical patients. Our hypothesis is that an early warning scoring system reduces the incidence of complications and mortality in hospitalized surgical patients.

## 2 Methods

This review is reported according to the Preferred Reporting Items for Systematic Reviews and Meta-Analyses (PRISMA) (Page et al., 2021). The protocol of this review is registered at PROSPERO, the international prospective register of systematic reviews, under the registration number CRD42018107799 and can be accessed online at https://www.crd.york.ac.uk/prospero/display_record.php?RecordID=107799.

### 2.1 Search strategy

To identify relevant publications, we conducted systematic searches in the bibliographic databases PubMed, Embase.com, CINAHL (Ebsco) and Wiley/Cochrane Library from inception up to June 20, 2023, in collaboration with a medical information specialist. The following terms were used (including synonyms and closely related words) as index terms or free-text words: “Early warning”, “Track-and-Trigger”, “Postoperative patients”.

The reference lists of the identified articles were searched for relevant publications. Duplicate articles were excluded by a medical information specialist using Endnote X20.0.1 (Clarivate^tm^), following the Amsterdam Efficient Deduplication (AED)-method (Otten et al., 2019) and the Bramer-method. (Bramer et al., 2016). All languages were accepted. The full search strategies for all databases can be found in Appendix A.

### 2.2 Selection process

Two reviewers (AS and YB) independently screened all potentially relevant titles and abstracts for eligibility. If necessary, the full text article was checked for the eligibility criteria. Differences in judgement were resolved through a consensus procedure that included consulting a third reviewer (PN). Studies were included if the effect of an early warning scoring system using spot check monitoring was studied in surgical patients. Studies performed on mixed medical-surgical wards were eligible for inclusion, if the proportion of surgical and medical patients were specified in the results. Eligible studies included randomized controlled trials and non-randomized studies of interventions, including before-and-after studies and quasi-randomized studies.

Studies were excluded if the efferent limb of a rapid response system (RRS) (e.g. medical emergency team or critical care outreach service) was the main study objective, or if continuous (remote) vital sign monitoring was used. In addition, pediatric, obstetric and intensive care studies were excluded. Certain publication types, such as editorials, legal cases, interviews and conference abstracts were also excluded.

### 2.3 Outcomes

Outcomes of interest were in-hospital mortality, cardiopulmonary arrests, unplanned intensive care admissions and postoperative complications.

### 2.4 Data extraction and quality assessment

The full text of the selected articles was obtained for further review. A data extraction sheet was used by two reviewers (AS and TWH) to register study design, study characteristics, patient characteristics, type of intervention, early warning scoring system and outcomes. Outcomes were reported with their corresponding effect sizes and p-values. Study quality was assessed by the same reviewers. If necessary, a third reviewer (PN) was consulted and disagreements were resolved by consensus. For the quality assessment of non-randomized studies (e.g. before-and-after studies) the ROBINS-I tool was used (Sterne et al., 2016). For cluster-randomized and parallel-group trials the version 2 of the Cochrane risk-of-bias tool for randomized trials (RoB 2 tool) was used (Higgins et al., 2011).

## 3 Results

**Fig. 1:**
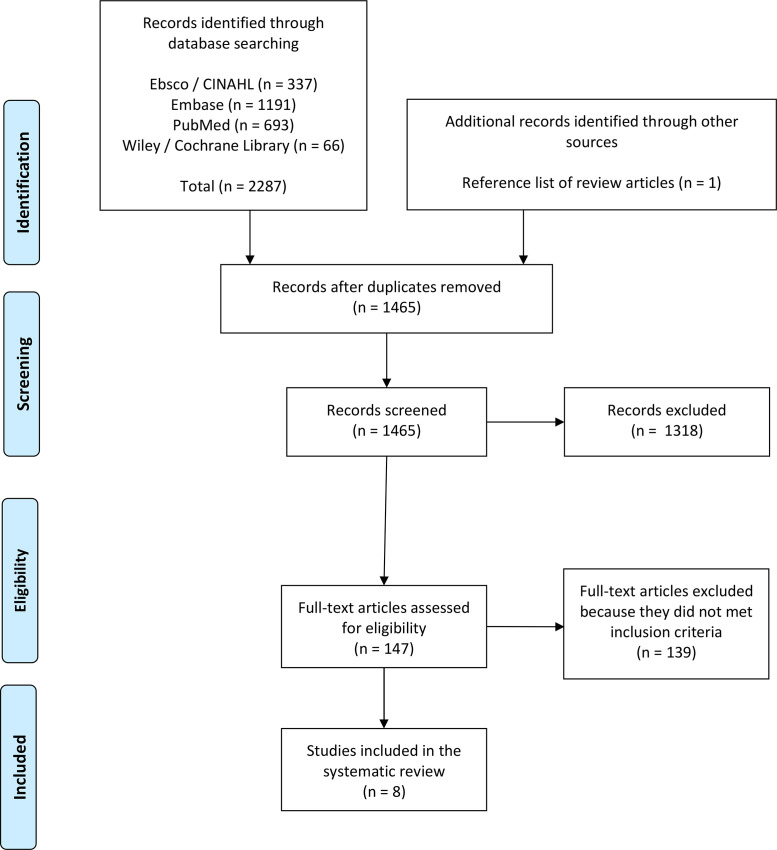
PRISMA flow diagram.

Through database searching, 2287 records were identified (Fig. 1). One additional record was identified after screening all reference lists. After removing duplicates, the title and abstract of 1465 articles were assessed. From 147 articles the full text was read, of which eight articles met the inclusion criteria for this systematic review.

### 3.1 Study characteristics

Study characteristics are shown in Table 1. All studies were published between 2010 and 2021. The studies consisted of six pre-post studies (Badr et al., 2021; Bunkenborg et al., 2014; De Meester et al., 2013; Heller et al., 2020; Mitchell et al., 2010; Patel et al., 2011), one stepped wedge cluster randomised controlled trial (Haegdorens et al., 2018) and one parallel-group randomised controlled trial (Kyriacos et al., 2019). There was great variation among studies in terms of study duration, varying from one month (Kyriacos et al., 2019) to seven years (Patel et al., 2011), and sample size, varying from 292 patients (Kyriacos et al., 2019) to 32,149 patients (Patel et al., 2011). Three studies only included surgical patients (De Meester et al., 2013; Heller et al., 2020; Patel et al., 2011). The proportion of surgical patients for the remaining five studies exceeded 50 % in all cases. In half of the studies, the surgical specialty of the included patients was not specified (Bunkenborg et al., 2014, Heller et al., 2020, Haegdorens et al., 2018, Kyriacos et al., 2019). Four studies reported the severity of co-existing diseases. The extent to which this was reported varied between studies. The methods used were past medical history (Badr et al., 2021), the Charlson Comorbidity Index (Haegdorens et al., 2018), the American Society of Anesthesiologist (ASA) classification of comorbidity, the New York Heart Association (NYHA) classification of heart insufficiency, Canadian Cardiovascular Society (CSS) classification of angina pectoris, German Diagnosis Related Group case weight (G-DRG; representing the relative resource utilization for patient care in comparison to national mean) (Heller et al., 2020) and presence of hypertension (Kyriacos et al., 2019).

**Table 1:** Study characteristics.

**Author, year**	**Study design**	**Type of ward**	**Study duration**	**Sample size**	**Patient characteristics;** **age (years), female (%) proportion surgical (%)**
Badr et al., 2021	Single center pre-post study	Mixed medical-surgical	6 months	Pre: *n* = 190 Post: *n* = 174	Pre: 40 ± 17, 38.5 % female, 78.4 % surgical Post: 43 ± 19, 27 % female, 80 % surgical
Bunkenborg et al., 2014	Single center pre-post study (including two postinterventional study periods)	Medical, surgical, an emergency admission ward and a high-intensity monitoring area	12 months	Pre: *n* = 1870 Post 1: *n* = 2079 Post 2: *n* = 2234	Pre: 58 ± 19, 58 % female, 79 % surgical Post 1: 57 ± 20, 56 % female, 79 % surgical Post 2: 57 ± 20, 59 % female, 79 % surgical
De Meester et al., 2013	Single center pre-post study	Surgical	10 months	Pre: *n* = 2359 Post: *n* = 1888	Pre: 58 [16–99], 53.4 % female Post: 58 [16–94], 49.6 % female
Haegdorens et al., 2018	Multi center stepped wedge cluster RCT	Medical and surgical	20 months	Control: *n* = 34.267 Intervention: *n* = 35.389	Control: 58.9 ± 18.6, 51 % female, 47.7 % surgical Intervention: 59.9 ± 18.2, 49 % females, 52.3 % surgical
Heller et al., 2018	Single center pre-post study	Surgical	24 months	Pre: *n* = 1896 Post: *n* = 1931	Pre: 64.6 ± 15.7, 36 % female Post: 65.2 ± 14.8, 35.5 % female
Kyriacos et al., 2019	Multi center parallel-group RCT	Medical and surgical	31 days	Control: *n* = 142 Intervention: *n* = 150	Control: 49.9 ± 17.5, 61.3 % female, 64.8 % surgical Intervention: 53.7 ± 16.2, 36 % female, 67.3 % surgical
Mitchell et al., 2010	Multi center pre-post study	Mixed medical-surgical	8 months	Pre: *n* = 1157 Post: *n* = 985	Pre: 58.6 ± 19.7, 44.3 % female, 52.2 % surgical Post: 57.4 ± 19.8, 45.2 % female, 50.6 % surgical
Patel et al., 2011	Single center pre-post study	Surgical	7 years	Total: *n* = 32.149 Number of participants per group NR	Total: age NR, 45 % female

Abbreviations: RCT **=** randomized controlled trial, NR **=** not reported.

### 3.2 Early warning scoring systems

The parameters of the early warning scoring systems were similar across studies, as shown in Table 2. In all studies systolic blood pressure (SBP), heart rate (HR), respiratory rate (RR), temperature and level of consciousness were part of the early warning scoring system . The majority of studies included oxygen saturation and oxygen therapy as well. Two studies included urine output (Mitchell et al., 2010; Patel et al., 2011) and one study noted team concern (Heller et al., 2020). In seven studies the early warning scoring system was based on previously developed and published early warning scoring system tools. In one study an unvalidated modified version of the early warning scoring system was implemented (Patel et al., 2011).

**Table 2:** Early Warning Scoring Systems interventions.

Author, year	EWSS parameters										Staff training	Participants	Components of training
	SBP	HR	RR	Temperature	Oxygen saturation	Oxygen therapy	AVPU	Sedation score	Team concern	Urine output			
Badr et al., 2021	●	●	●	●	●	●	●				Yes	Nursing staff	Two months training utilizing: lecture, group discussion, and clinical scenarios
Bunkenborg et al., 2014	●	●	●	●			●				Yes	a; Nursing staff (2009–2010)b; Nursing staff (2010–2011)c; Physiciansd; Senior nursing and medical staff	a; 4-hour teaching session and a 4-hour simulation training b; 2-hour teaching sessionc; 30- to 45-minute teaching sessiond; Three 1-hour knowledge-sharing session
De Meester et al., 2013	●	●	●	●	●		●				Yes	Nursing staff	Training sessions during one month
Haegdorens et al., 2018	●	●	●	●	●	●	●				Yes	Nursing staff	4-hour interactive training
Heller et al., 2018	●	●	●	●	●	●	●		●		Yes	Medical staff	Intense training during the first 2-month study period
Kyriacos et al., 2019	●	●	●	●	●	●	●				Yes	Nursing staff	2-hour face-to-face training
Mitchell et al., 2010	●	●	●	●	●			●		●	Yes	Nursing staff Allied health care workers Medical officers	E-learning package, and; 3-hour face-to-face low fidelity simulation package
Patel et al., 2011	●	●	●	●			●			●	NR	NR	NR

Abbreviations: EWSS **=** Early Warning Scoring Systems, SBP **=** systolic blood pressure, HR **=** heart rate, RR **=** respiratory rate, AVPU **=** alert verbal pain unresponsive scale, NR **=** not reported.

In addition to the early warning scoring system , all studies implemented a response protocol with dedicated actions based on the early warning scoring system results. Two studies implemented the SBAR communication protocol (Haegdorens et al., 2018; Kyriacos et al., 2019) as an additional component of the intervention. Apart from one study, early warning scoring system measurements were performed intermittently and manually (Heller et al., 2020). Training specifics varied between studies (Table 2).

### 3.3 Risk of bias

The results of the risk of bias assessment are shown in Table 3. None of the studies reported that the data were analyzed in accordance with a pre-specified analysis plan. Both randomized studies were classified as high risk of bias because the implementation of the interventions was not blinded (Haegdorens et al., 2018; Kyriacos et al., 2019). Four non-randomized studies were categorized as serious risk of bias (Badr et al., 2021; De Meester et al., 2013; Heller et al., 2020; Mitchell et al., 2010) and two as critical risk of bias (Bunkenborg et al., 2014; Patel et al., 2011). Regarding confounding, two non-randomized studies were scored as critical because no confounders were included in the analyses (Bunkenborg et al., 2014; Patel et al., 2011). Of the four other non-randomized studies, two did not report the results of regression analysis (Badr et al., 2021; Mitchell et al., 2010). One study adjusted for several characteristics but not for comorbidity (De Meester et al., 2013)*,* and one study adjusted for G-DRG case weight, as a derivative of comorbidity (Heller et al., 2020).

**Table 3:** Risk of bias assessment.

## 4 Outcomes

### 4.1 In-hospital mortality

A summary of study outcomes is shown in Table 4. All studies reported postoperative in-hospital mortality. Mortality rates varied between 0.1 % and 3.7 %. In three studies mortality was statistically significantly lower in the intervention group (Bunkenborg et al., 2014; De Meester et al., 2013; Mitchell et al., 2010). One study performed a multiple regression analysis, but did not adjust for comorbidity as a confounder, and reported a relative risk reduction of 73.7 % (95 % CI 22.8–91.0, *p* = 0.015) for 6-day postoperative mortality (De Meester et al., 2013). In the five other studies no statistically significant decrease in mortality was shown after implementation of an early warning scoring system.

**Table 4:** Outcomes.

Author, year	Outcomes (intervention versus control period)			
	In-hospital mortality	Cardiopulmonary arrests	Unplanned ICU-admissions	Complications
Badr et al., 2021	2 (1.1 %) vs. 7 (3.7 %), OR 0.29 (95 % CI 0.08–1.1), *p* = 0.120*	2 (1.1 %) vs. 9 (4.7 %), OR= 0.23 (95 % CI 0.00–0.98), *p* = 0.046	3 (1.7 %) vs. 10 (5.3 %), OR= 0.32 (95 % CI 0.0–7–1.3), *p* = 0.049	*Emergency surgery:* 0 (0 %) vs. 12 (6.3 %), OR= 0 (95 % CI 0–0.4), *p* = 0.001 *Acute renal failure:* 2 (1.1 %) vs. 13 (6.8 %), OR= 0.16 (95 % CI 0.03–0.65), *p* = 0.006
Bunkenborg et al., 2014	5 (0.2 %) vs. 13 (0.7 %), 17 vs. 61 per 100 adjusted patient years, *p* = 0.013*	3 (0.13 %) vs. 7 (0.4 %), p-value NR	17 (0.8 %) vs. 17 (0.9 %), p-value NR	
De Meester et al., 2013	*6-day postoperative mortality:* 4 (0.2 %) vs. 19 (0.8 %), adjusted RRR^#^ 73.7 % (95 % CI 22.8–91.0), *p* = 0.015*			*Length of hospital stay (days):* 4.11 (95 % CI 3.92–4.30) vs. 4.55 (95 % CI 4.34–4.76), *p* = 0.004 *6-day postoperative resurgery:* 78 (4.1 %) vs. 141 (6 %), adjusted RRR^#^ 30.9 % (95 % CI 9.5–47.2), *p* = 0.007
Haegdorens et al., 2018	23 (0.06 %) vs. 52 (0.15 %), model 1 adjusted OR= 0.82 (95 % CI 0.34–1.95), model 2 adjusted OR= −0.00023 (95 % CI −0.00128–0.00083), *p*= NS*	35 (0.1 %) vs. 46 (0.13 %), model 1 adjusted OR= 0.71 (95 % CI 0.33–1.52), model 2 adjusted OR= 0.54 (95 % CI 0.18–1.64), *p*= NS	363 (1.03 %) vs. 224 (0.65 %), model 1 adjusted OR= 1.23 (95 % CI 0.91–1.65), model 2 adjusted OR= 1.24 (95 % CI 0.84–1.83), *p*= NS	
Heller et al., 2018	8.8 vs. 8.5 (expressed as deaths 1000 German DRG case weight points), *p*= NS	4 (0.2 %) vs. 10 (0.5 %), German DRG case weight adjusted analysis, *p*= <0.001	58 (3 %) vs. 69 (3.6 %), German DRG case weight adjusted analysis, *p*= <0.001	*Length of hospital stay (days):* 13.8 vs. 14.7, German DRG case weight adjusted analysis, *p*= NS
Kyriacos et al., 2019	4 (2.7 %) vs. 1 (0.7 %), OR= 3.89 (95 % CI 0.43–35.23), *p* = 0.37*		1 (0.7 %) vs 0 (0 %), OR NR	*Prolonged hospitalization:* 0 (0 %) vs. 2 (1.4 %), OR NR
Mitchell et al., 2010	2(0.2 %) vs. 11 (1.0 %), RRR^$^= 1.57 (95 % CI 1.24–1.99), *p* = 0.03* Total mortality: 6 (0.6 %) vs. 30 (2.6 %), RRR^$^= 1.56 (95 % CI 1.34–1.81), *p*= <0.001		5 (0.5 %) vs. 21 (1.8 %), RRR^$^= 0.28 (95 % CI 0.11–0.74), *p* = 0.005	*Hospital length of stay (days):* 4.8 vs. 4.0, RRR^$^= 0.02, 95 % CI, *p* = 0.03 *Acute myocardial infarction:* 2 (0.2 %) vs. 1 (0.1 %), RRR^$^= 0.60, 95 % CI and p-value NS *Pulmonary embolus:* 2 (0.2 %) vs. 4 (0.4 %), RRR^$^= 0.69, 95 % CI and p-value NS *Respiratory failure:* 1 (0.1 %) vs. 7 (0.6 %), RRR^$^= 0.08, 95 % CI and p-value NS *Acute renal failure:* 0 (0.0 %) vs. 1 (0.1 %), RRR^$^= 1, 95 % CI and p-value NS
Patel et al., 2011	2.29 % vs. 3.22 %; decrease of 0.9 % (95 % CI 0.53–1.31), *p* = 0.092			

Abbreviations: OR = odds ratio, CI = confidence interval, NR = not reported, RRR^#^ = relative risk reduction, NS = not significant, German DRG = German Diagnosis Related Group, RRR^$^ = relative risk ratio.

⁎ Death was defined as death without the presence of any form of a 'Do Not Attempt Resusiscitation' (DNAR) order.

### 4.2 Cardiopulmonary arrests

In four studies, the incidence of cardiopulmonary arrest was reported, which was ≤ 0.5 % in three studies in both intervention group and control group (Bunkenborg et al., 2014; Heller et al., 2020; Haegdorens et al., 2018). In two of the four studies a statistically significant decrease in cardiopulmonary arrests was found in the intervention groups (Badr et al., 2021; Heller et al., 2020), of which one study adjusted for a derivative for comorbidity and reported 0.2 % cardiopulmonary arrests in the intervention group versus 0.5 % arrests in the control group (*p* = <0.001) (Heller et al., 2020).

### 4.3 Unplanned ICU-admissions

Two out of eight studies did not report data with regard to unplanned ICU-admissions. Half of the studies who did, reported statistically significantly lower rates of post-operative unplanned ICU admissions in the intervention groups (Badr et al., 2021; Heller et al., 2020; Mitchell et al., 2010), of which one was adjusted for confounding (Heller et al., 2020). In contrast, two studies reported higher rates of unplanned ICU admissions in the intervention group, but this difference was not statistically significant (Haegdorens et al., 2018; Kyriacos et al., 2019). In one study the rate of ICU-admission was similar in the intervention group and control group (Bunkenborg et al., 2014).

### 4.4 Complications

The studies are heterogeneous regarding the types of complications described. In four studies the length of hospital stay was reported. In one study, hospital stay was lower in the intervention group (De Meester et al., 2013) in contrast to another study which reported a longer hospital stay (Mitchell et al., 2010). Two studies found no decrease in length of hospital stay (Heller et al., 2020; Kyriacos et al., 2019). One study reported a statistically significant decrease in emergency surgery (Badr et al., 2021), one study in reoperation after six days (De Meester et al., 2013), and one study in acute renal failure (Badr et al., 2021).

### 4.5 Adherence to intervention

Only two out of eight studies reported the percentage of staff members who attended early warning scoring system training. For both studies this percentage was high; i.e. above 77 % (Mitchell et al., 2010; Kyriacos et al., 2019). Three studies did not report data regarding the frequency of vital sign measurements. Five studies showed higher frequency of vital sign measurements in the intervention groups (Badr et al., 2021; De Meester et al., 2013; Mitchell et al., 2010; Haegdorens et al., 2018; Kyriacos et al., 2019).

## 5 Discussion

This systematic review studied the effect of the implementation of an early warning scoring system on mortality and complications in surgical patients. The evidence in favor of an early warning scoring system in view of postoperative outcomes in surgical patients is low, primarily due to a limited number of studies and poor study design. Only two randomized controlled trials were included in this review and the remaining studies had a pre-post design, which were classified as serious risk of bias or worse. Out of eight studies only three studies examined the effect of an early warning scoring system implementation in the specific surgical population. After implementation of an early warning scoring system , a statistically significant decrease in mortality was seen in three studies. In two studies a decrease in cardiopulmonary arrests was reported and in three studies a decrease in ICU-admissions. The studies were heterogeneous regarding the types of complications reported.

Several studies have examined the predictive accuracy of an early warning scoring system to distinguish between the patients who will develop a poor outcome (in means of an area under the curve (AUC)), mainly in the emergency department setting. In 2021 a systematic review was published which studied the predictive performance of an early warning scoring system in different clinical settings. Based on nine studies with a surgical setting, that review calculated a mean AUC of 0.80 for mortality, 0.79 for ICU-admission and 0.75 for cardiac arrest (Alhmoud et al., 2021). Besides predictive accuracy, the effect of an early warning scoring system implementation on patient outcomes is important. In 2021 Cochrane published an update of the previously published review on the effect of an early warning scoring system and rapid response system implementation on acute hospital wards (McGaughey et al., 2021). The conclusion of the Cochrane review is that there is low-certainty evidence that an early warning scoring system and rapid response system may lead to differences in patient outcomes. To our best knowledge there are no reviews available which study the effect of the implementation of an early warning scoring system on patient outcomes in the surgical population. Despite a good predictive performance of early warning scoring systems and widespread implementation, the impact on patient outcomes remains to be determined.

This review has several limitations. First, the limited number of included studies were of poor quality. It is notable that few studies considered confounding of comorbidity in the analysis. However, it should be mentioned that both randomised controlled trials were scored as high risk of bias because of the unblinded implementation of an EWSS, which seems unavoidable. Second, the studies were for the most part similar in terms of early warning scoring system parameters and the response protocols based on the early warning scoring system scores. However the training programs of staff differed between the studies and little data was available regarding the compliance to the training programs. Third, in six out of eight studies mortality was defined as ‘death without a do not attempt resuscitation (DNAR) order’. Therefore, the results should be extrapolated with caution to the general surgical population.

One important shortcoming of early warning scoring systems is that vital signs are measured intermittently. This is one of the reasons that there is increasing interest in the implementation of continuous remote vital signs monitoring for the early detection of patient deterioration on the ward. Nevertheless, remote monitoring systems can produce high rates of false-positive alarms that the nursing staff is expected to interpret, potentially leading to alarm fatigue (Leenen et al., 2022). Due to higher costs and staff shortages, it is not yet feasible to implement remote monitoring systems on a large scale on general wards. Therefore early warning scoring systems will remain of great importance on the wards in the coming years. As a consequence, it is still important to properly investigate whether an early warning scoring system actually improves patient outcomes in the surgical setting. Future research needs to focus on well-designed randomised controlled trials conducted in the surgical population.

## Conclusions

Overall, the evidence in favor of an early warning scoring system to reduce complications and mortality in surgical patients is low, mainly due to a limited number of studies and poor study design. Well-designed trials are needed to investigate whether an early warning scoring system improves outcome in surgical patients.

## Funding

No external funding.

## CRediT authorship contribution statement

**Annick Stolze:** Writing – original draft, Visualization, Project administration, Methodology, Investigation, Formal analysis, Data curation, Conceptualization. **Tara N.M. Woolley-Hendriks:** Writing – original draft, Visualization, Investigation. **Yara Bassa:** Investigation. **Ralph de Vries:** Methodology, Formal analysis. **Christa Boer:** Writing – review & editing, Supervision. **Peter G. Noordzij:** Writing – original draft, Supervision, Methodology, Conceptualization.

## Declaration of competing interest

None.

## Appendix Supplementary materials

Image, application 1
