# Supplementary material for: The effect of early warning scoring systems on adverse outcome in surgical patients: A systematic review
Source: Int J Nurs Stud Adv. 2024 Oct 28;7:100256. doi: 10.1016/j.ijnsa.2024.100256 (PMC11665940; doi:10.1016/j.ijnsa.2024.100256)
Supplement: Supplementary file 1 [file mmc1.docx]

**Appendix A**

**PubMed Session Results (20 Jun 2023)**

[tw] = includes all words and numbers in the title, abstract, other abstract, MeSH terms, MeSH Subheadings, Publication Types, Substance Names, Personal Name as Subject, Corporate Author, Secondary Source, Comment/Correction Notes, and Other Terms

[tiab] = words in title or abstract or author keywords

| **#** | **Query** | **Results** |
| --- | --- | --- |
| #3 | #1 AND #2 | 693 |
| #2 | postoperati*[tw] OR "post-operati*"[tw] OR postsurg*[tw] OR "post-surg*"[tw] OR surgical[tw] OR surgery[tw] | 3,710,918 |
| #1 | "Early Warning Score"[Mesh] OR "early warning*"[tiab] OR (EWS[tiab] AND warning*[tiab]) OR MEWS[tiab] OR SEWS[tiab] OR "Track-and-Trigger"[tiab] OR "warning system*"[tiab] OR "warning score*"[tiab] | 11,722 |

**Embase.com Session Results (20 Jun 2023)**

/exp = EMtree keyword with explosion

:ab,ti,kw,de = words in abstract or title or author keywords or index terms

| **#** | **Query** | **Results** |
| --- | --- | --- |
| #3 | #1 AND #2 | 1,191 |
| #2 | postoperati*:ab,ti,kw,de OR 'post-operati*':ab,ti,kw,de OR postsurg*:ab,ti,kw,de OR 'post-surg*':ab,ti,kw,de OR surgical:ab,ti,kw,de OR surgery:ab,ti,kw,de | 4,444,132 |
| #1 | 'early warning system'/exp OR 'early warning score'/exp OR 'early warning*':ab,ti,kw OR (ews:ab,ti,kw AND warning*:ab,ti,kw) OR mews:ab,ti,kw OR sews:ab,ti,kw OR 'track-and-trigger':ab,ti,kw OR 'warning system*':ab,ti,kw OR 'warning score*':ab,ti,kw | 15,189 |

**Ebsco / CINAHL Session Results (20 Jun 2023)**

MH = index terms

TI = words in title

AB = words in abstract
SU = subject heading

| **#** | **Query** | **Results** |
| --- | --- | --- |
| S3 | S1 AND S2 | 337 |
| S2 | TI (postoperati* OR "post-operati*" OR postsurg* OR "post-surg*" OR surgical OR surgery) OR AB (postoperati* OR "post-operati*" OR postsurg* OR "post-surg*" OR surgical OR surgery) OR SU (postoperati* OR "post-operati*" OR postsurg* OR "post-surg*" OR surgical OR surgery) | 791,350 |
| S1 | (MH "Early Warning Score") OR TI ("early warning*" OR (EWS AND warning*) OR MEWS OR SEWS OR "Track-and-Trigger" OR "warning system*" OR "warning score*") OR AB ("early warning*" OR (EWS AND warning*) OR MEWS OR SEWS OR "Track-and-Trigger" OR "warning system*" OR "warning score*") OR SU ("early warning*" OR (EWS AND warning*) OR MEWS OR SEWS OR "Track-and-Trigger" OR "warning system*" OR "warning score*") | 3,405 |

**Wiley / Cochrane Library Session Results (20 Jun 2023)**

:ab,ti,kw = words in abstract or title or author keywords

| **#** | **Query** | **Results** |
| --- | --- | --- |
| #3 | #1 AND #2 | 66 |
| #2 | postoperati*:ab,ti,kw or (post NEXT operati*):ab,ti,kw or postsurg*:ab,ti,kw or (post NEXT surg*):ab,ti,kw or surgical:ab,ti,kw or surgery:ab,ti,kw | 331,861 |
| #1 | (early NEXT warning*):ab,ti,kw or (EWS:ab,ti,kw and warning*:ab,ti,kw) or MEWS:ab,ti,kw or SEWS:ab,ti,kw or "Track-and-Trigger":ab,ti,kw or (warning NEXT system*):ab,ti,kw or (warning NEXT score*):ab,ti,kw | 544 |
